# Exploring considerations for becoming a GP practice owner: a qualitative study

**DOI:** 10.3399/BJGPO.2024.0213

**Published:** 2025-07-30

**Authors:** Hinda A Stegeman, Manna A Alma, Hanneke PM Vervoort, Vivian van Vliet, Nynke D Scherpbier, Daniëlle MC Jansen, Marjolein Y Berger

**Affiliations:** 1 Business Administration, Primary and Long Term Care, University Medical Centre Groningen, Groningen, The Netherlands; 2 Applied Health Research, University Medical Centre Groningen, Groningen, The Netherlands; 3 Social Psychology, Primary and Long Term Care, University Medical Centre Groningen, Groningen, The Netherlands; 4 General Practice, Primary and Long Term Care, University Medical Centre Groningen, Groningen, The Netherlands; 5 Sociology, Primary and Long Term Care, University Medical Centre Groningen, Groningen, The Netherlands

**Keywords:** practice organisation, qualitative research, continuity of patient care, general practice, general practitioners, primary healthcare

## Abstract

**Background:**

General practice owners are responsible for access to care 24 hours a day, but they can struggle to find associates or successors. Fewer practice owners means that the core value, continuity of care (COC), is at risk. However, little is known about the career considerations of young GPs and barriers and facilitators to become practice owners.

**Aim:**

To explore the considerations of GPs for becoming a practice owner.

**Design & setting:**

A qualitative study of GP trainees, freelance and salaried GPs, practice owners, and ex-practice owners in the north of The Netherlands.

**Method:**

Ninety GPs were purposively recruited for focus groups and interviews, which were audio and video-recorded, transcribed verbatim, and analysed thematically.

**Results:**

Becoming a practice owner results from a complex interplay between professional, personal, external, and process-related factors, often over an extended period. Participants indicated that COC, autonomy, and personal development had predominantly positive impacts on decisions about practice ownership. Factors that negatively affected considerations included work–life balance, ultimate responsibility, negative role models, unappealing practices, (un)preparedness, and issues with the process. Of note, non-practice owners felt that practice ownership could not be discussed.

**Conclusion:**

Modifications to perceived behavioural control and subjective norms are needed, together with an open dialogue among GPs about practice ownership and alternative models. Our findings offer a foundation for further prospective quantitative research into efforts designed to address the shortage of practice owners in The Netherlands and other countries. This could uncover universal and country-specific themes.

## How this fits in

Existing literature has identified three enablers of practice ownership, of which we could only confirm one (autonomy) and add another two (continuity of care [COC] and personal development). We could confirm three of the five barriers identified in the earlier research (unpreparedness, work–life balance, and ultimate responsibility), and could add another three (lack of role models, unavailability of appropriate practices, and process and approach features). In addition, we identified the following new considerations that have not been mentioned previously, which are not necessarily enablers or barriers: social responsibility; task-related self-efficacy; settling in a region; and legislation. By combining our results with the theory of planned behaviour, we propose strategies that can empower GPs to make informed decisions about practice ownership and provide suggestions for future research.

## Introduction

Strong primary care has a positive effect on the health of the entire population, with the provision of person-focused care over time being a key feature.^1^ This continuity of care (COC) is associated with lower mortality^2,3^ and fewer hospital admissions,^4–6^ but it depends on a long doctor–patient relationship.^1,7,8^ Easy access to primary care with COC is particularly important in rural areas with low economic statuses, where it has been shown to increase the health status of the population.^9,10^

Unfortunately, the features that constitute strong primary care are under threat in many countries worldwide. In high-income countries, not only does the population live for much longer, but also older people need to live at home for much longer, causing an ever-greater shift in healthcare provision from secondary to primary care.^7^ Consequently, primary care must manage increasingly complex and labour-intensive care needs that require a deeper knowledge of disease states^7^ and the time and competence to collaborate effectively with other care and welfare entities.^7,11,12^ These changes have been associated with increased GP staffing levels in The Netherlands over the past two decades, rising from 2.1 full-time staff per practice in 2001 to 5.2 in 2020. In parallel, there has been a necessary increase in personnel and building costs,^7,9^ the allocation of more time, and a need for entrepreneurial skills in personnel and financial management.^9^

In The Netherlands, the GP is the key figure in primary care. Every Dutch citizen is registered in a general practice that offers free medical care to registered patients, with the cost of that care being covered by insurance companies.^8^ A GP has traditionally been the practice owner and has had 24-hour responsibility for patient access to care.^9,13^ Given the challenges of modern care, however, it is not surprising that many young Dutch GPs are reluctant to become practice owners. This is evidenced by a decrease in the percentage of GP practice owners from 87% in 2012 to 64% in 2022,^14–16^ accompanied by an increase in freelance and salaried GPs from 16% of all GPs in 2000 to 39% in 2019.^12,13,15^ An increasing number of GP practices are also being taken over by commercial businesses.^17^

These trends have had a major impact on healthcare access and COC in the north of The Netherlands,^18–20^ contributing to a possible deterioration in the health of a population that has a low economic status and is ageing rapidly.^9,10^ The shortage of practice owners has been felt even harder in this area, which is home to five of the least popular regions to settle as a practice owner. More than 39% of existing practice owners in two northern provinces have struggled to find associates or successors, a figure that substantially exceeds the national average of 25%.^21,22^

Little is known about the career considerations of young GPs, particularly regarding their decisions about practice ownership. Previous literature from Australia and England reported barriers included insufficient knowledge and skills about practice ownership,^23–26^ workload and work–life balance,^23–25,27^ financial concerns,^23–25,27^ increased responsibility,^25,27^ and bureaucracy.^23^ Reported enablers included flexibility,^25^ autonomy, a desire to be self-employed and establishing one’s own vision,^25^ and financial reward.^25^ Given the differences in healthcare systems and practice sizes between the countries,^7,9,13,28–30^ it is unclear whether these factors are relevant in The Netherlands.

To ensure COC, especially in underserved or rural areas, understanding the career choices and motivations of GPs is crucial. Given the reliance of the Dutch healthcare system on GP-owned practices to provide COC, we are particularly interested in decisions about practice ownership. Therefore, this qualitative study aims to explore the considerations and motivations of GPs when deciding whether to become a practice owner.

## Method

### Study design

We conducted a qualitative study of the personal experiences, perspectives, and career choices of GPs and practice owners. We began with focus group discussions (FGDs) to gather a broad spectrum of considerations. Individual semi-structured interviews were conducted with a few participants to provide more in-depth insights. Data collection continued until we reached data saturation and no new codes emerged. Our study adhered to the Consolidated criteria for reporting qualitative research (COREQ) guidelines for processing and presenting the results.^31^

### Sampling and recruitment

We purposively sampled GPs at different career stages and had separate FGDs with each group (Supplementary Box S1). The groups were as follows: (1) group of GPs in their first and last years of training; (2) freelance and salaried GPs who cannot or do not want to be a practice owner yet; (3) GPs who became a practice owner in the 4 years before this study; and (4) ex-practice owners who resigned early, before reaching age 50 years. Freelance and salaried GPs and ex-practice owners took part in additional interviews.

After three FGDs with freelance and salaried GPs, it became apparent that we lacked the perspectives of GPs who were highly uncertain about becoming a practice owner, as well as ex-practice owners and those with experience of failed practice takeovers. To address this, we issued a second call for participation, explicitly seeking individuals with these perspectives.

Participants were recruited through newsletters, targeted emails, and Facebook groups associated with GP care organisations in the north of The Netherlands, as well as from the GP specialty training programme in Groningen. We also employed snowball sampling. Participants could express an interest in our study and specify whether they preferred to participate in an FGD, an interview, or had no preference. Before their FGD or interview, participants were asked to complete a demographic questionnaire. Freelance and salaried GPs, practice owners, and ex-practice owners received a €50 (approximately 42 GBP) gift voucher. GPs in training participated during training hours and did not receive a gift voucher.

### Data collection

The FGDs and interviews followed a semi-structured approach with a customised topic list (Supplementary Box S2). FGD topics were based on literature about career development.^32,33^ New topics emerging during FGDs were added to the topic list. The FGDs were facilitated by an experienced qualitative researcher (MA) and observed by HS, and the interviews were conducted by HS. Between December 2019 and March 2021, we conducted four FGDs and one interview in-person, plus another 10 FGDs and five interviews online (via Microsoft Teams, owing to COVID-19 restrictions). FGDs lasted 105–120 minutes, and interviews lasted 45–90 minutes. After obtaining participant consent, all FGDs and interviews were video- and audio-recorded and transcribed verbatim.

### Data analyses

We followed the Braun and Clarke phases for thematic analysis.^34^ Table 1 details the steps. Initially, five researchers read the transcripts of three FGDs of different subgroups and noted initial ideas for coding. These codes were defined collectively and compiled into a codebook using ATLAS.ti 21. HS then coded the remaining data and this was reviewed by one of the other researchers. Any new codes were defined and cross-checked by the team before inclusion in the codebook. After completing the coding for all FGDs and interviews, five researchers took part in peer debriefing. Thematic analysis was conducted using mind-mapping techniques to develop themes. Findings were discussed among all researchers to establish the essence, definition, and names of each theme. Illustrative quotes were translated by JV and a native English speaker.

**Table 1.** Thematic analysis process using phases of Braun and Clarke

Phase	Action	Result
1. Familiarising with data	Reading three transcripts of different subgroups (HS, MA, HV, VvV, SdV)	Individual initial list of ideas for coding
2. Generating initial codes	a. Jointly going through transcripts of three focus groups, focusing on discussing everyone’s initial ideas of codes and arriving at a corresponding idea (HS, MA, HV, VvV, SdV)	Initial codebook with definitions of the codes
	b. Coding of the remaining data by HS, reviewed transcript by one of the other researchers (MA, HV, VvV, SdV). New codes were defined and cross-checked by the whole team before inclusion in the codebook	Final codebook
	c. Peer debriefing, looking back on the process and cooperation, and sharing the overall impression of the data (HS, MA, HV, VvV, SdV)	Shared overall view across the data
3. Searching for themes	a. Developing themes using mind mapping (HS and MA)	Initial concept of themes
	b. Discussion of overarching themes (HS, MA, HV, VvV, SdV)	Concept of themes
4. Reviewing themes	Discussion of the themes with the authors of the article (HS, MA, HV, VvV, DJ, MB)	Completion of the thematic map
5. Defining and naming themes	Writing down the essence of the themes by HS, reviewed by authors (MA, DJ, HV, VvV, MB)	Completion definition of themes; concept of result paragraph of article
6. Producing the report	Rewriting the results in several stages (HS), always commented by the other authors (MA, DJ, HV, VvV, NS, MB). During this process, a synthesis was formulated and discussed with the authors (MA, DJ, HV, VvV, NS, MB)	Finalised text of the results section

### Reflexivity

The authors formed an interprofessional research group with expertise in general practice (MB, NS, VvV), social sciences (DJ, MA, JV), and public and business administration (SdV, HS). HS, NS, DJ, and SdV had experience in primary care organisation, with HS and SdV working at a support organisation for primary care. HS and MB initiated this study after a discussion about a practice ownership survey led by GPs and HS’s organisation. The outcomes highlighted broadly stated barriers (for example, work–life balance, administrative tasks) that required deeper investigation. MA and DJ contributed research expertise, while NS, MY, and VvV were involved in GP specialty training.

## Results

**Figure 1.**
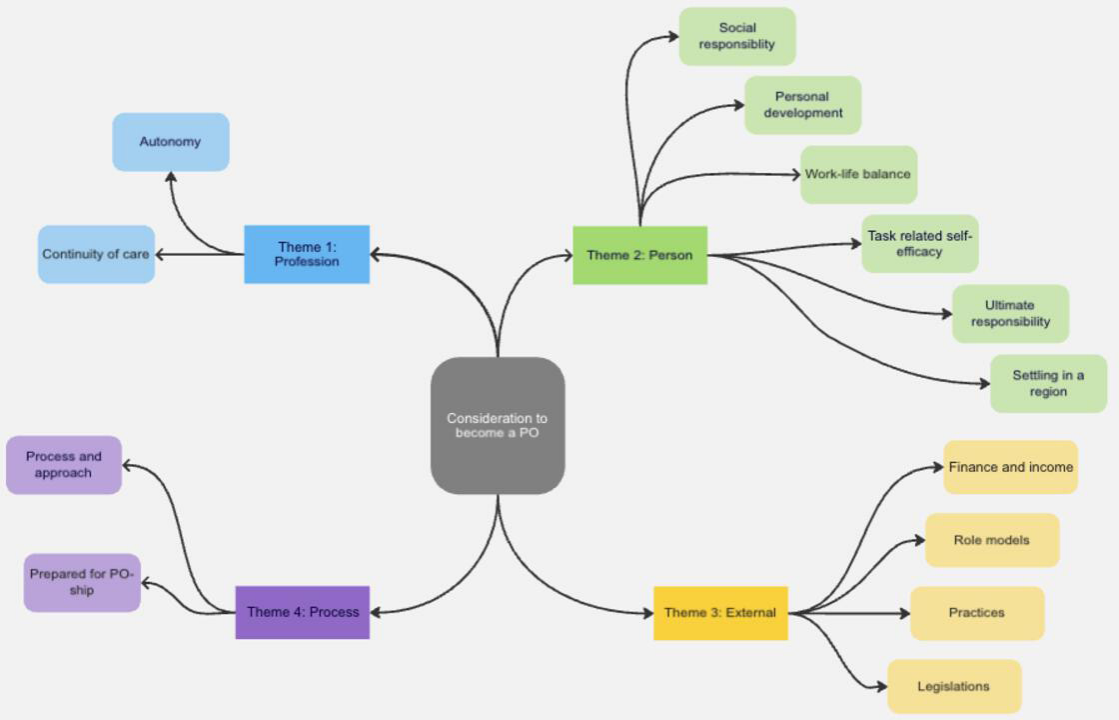
Themes and sub-themes. PO = practice owner

Participant demographics are summarised in Table 2. Thematic analysis revealed four themes, namely profession, personal, external, and process that each contained sub-themes (Figure 1). Themes and representative quotes are presented in Supplementary Table S1.

**Table 2.** Background information of participants

		GP trainees (*n* = 41)	Freelance and salaried GPs (*n* = 31)	POs (*n* = 12)		Freelance and salaried GPs (*n* = 3)	Ex-POs^a^ (*n* = 3)
**Number of FGDs** **(14 total)**		5	6 (1 mixed group with 1 ex-PO)	3	**Number of Interviews (6 total)**	3	3
**Sex**	Male/female	14/27	5/26	1/11		1/2	2/1
**Age in years**	Range, years	26–38	31–47	33–44		36–51	46–48
	Mean	30.9	36.8	38.7		43.3	47
	Missing	*n* = 2	-	-		-	*n* = 1
**Years after graduation as GP**	Range	-	0–15	2–17		3–16	9–17
	Mean		5.58	8.9		10	13
	Missing	-	-	*n* = 1		-	-
**Children**	Yes/no	14/25	23/8	10/1		3/0	2/0
	Range (number of children)	1–4	1–4	1–7		2–3	2–3
	Mean (if children yes)	1.71	2.17	3.1		2.3	2.5
	Missing	*n = 2*	-	*n* = 1		-	*n* = 1
**Children’s age in years**	Range (years)	0–10	0–17	0–16		3–18	10–19
	Mean	3.2	6.1	6.2		10.7	12.6
	Missing	-	-	-			*n* = 1

^a^Practice owners who resigned early, before reaching age 50 years. FGD = focus group discussion. PO = practice owners

### Theme 1: Profession

#### Continuity of care

COC is an important value in career choices, whether becoming a GP or a practice owner. Non-practice owners (that is, GP trainees, freelance and salaried GPs) expect that practice owners perceive more COC (that is, a long-term relationship with patients and personnel), leading to better care and a pleasant way of working. However, some freelance and salaried GPs can establish COC if they work at one practice for a long time; for this group, COC was not a key consideration for becoming a practice owner. Ex-practice owners found it challenging to resign early owing to the loss of COC.

#### Autonomy

Autonomy played a role in career choices. Non-practice owners anticipate that practice owners enjoy greater professional autonomy in implementing their vision of GP care and personal development, leading to increased job satisfaction. This was confirmed by practice owners. Notably, some ex-practice owners experienced too little autonomy in collaborating with neighbouring practices and faced constraints owing to health insurer regulations, leading to their early resignation.

### Theme 2: Person

#### Social responsibility

Taking social responsibility for GP care appears to be a positive factor for becoming a practice owner. Social responsibility refers to doing what is right, fulfilling the social norm of being a practice owner, feeling fully accepted by colleague practice owners, and preventing commercial takeovers of GP practices. However, non-practice owners feel societal pressure owing to older practice owners’ expectations and perceive judgement for any hesitation; they report finding it a challenge to discuss questions about practice ownership. Although they feel responsibility for GP care, they commented that this is not seen by practice owners. Some participants, both practice owners and non-practice owners, noted that non-practice owners feel as if they are not fully accepted and valued. However, both groups want open dialogue to establish a sustainable organisation of GP care and the role that practice ownership has in it.

#### Personal development

In the first years after graduation, GPs focus on professional development by working with different populations and practices, mostly as a freelance GP. Freelance and salaried GPs mentioned that they would look for other challenges at some point. Practice ownership is often the primary focus, but they also noted alternative career paths, such as teaching, research, and shaping GP care policies. Non-practice owners expected that practice owners have more opportunities to develop management skills and specialisation as a GP.

#### Work–life balance

Non-practice owners fear that practice owners have a poor work–life balance and anticipate a higher workload and less time for family and hobbies. They also find that practice owners are less flexible in choices about their work–life balance and taking time off, which was confirmed by some ex-practice owners who resigned owing to concerns about their work–life balance. However, practice owners did report more flexibility than expected beforehand. Non-practice owners expect to have more career flexibility and time to consider practice ownership as their children grow older.

#### Task-related self-efficacy

Many non-practice owners believe they have the capacity to become a practice owner, but some have low self-efficacy owing to not seeing themselves as entrepreneurs, or setting high standards, or struggling with decision making. A few considered partnership an option. However, lack of managerial skills led one ex-practice owner to resign early.

#### Ultimate responsibility

Most participants preferred not to have sole responsibility and were attracted to practice owner roles if they can share responsibility with one to three other GPs or collaborate with neighbouring practices. A barrier to becoming a practice owner was finding the right partner(s). Practice owners highlighted the practicality and comfort of shared responsibility and stressed the importance of compatible, complementary practice co-owners willing to invest time and compromise. An inability to cooperate effectively with neighbouring practices led some ex-practice owners to resign.

#### Settling in a region

Finally, desires to settle in a specific location affected career decisions and were a prerequisite for becoming a practice owner. This was often contingent on the employment prospects of the GP’s partner, which was prioritised because it was felt that GPs can find work easily throughout The Netherlands.

### Theme 3: External

#### Finance and income

Although ideas about finances and income differed, this was not an important consideration for participants in becoming a practice owner. Some noted that it can be time-consuming to find a practice to share with a partner that generates ample income for both. One ex-practice owner mentioned inadequate finances for a new practice location as a reason for quitting.

#### Role models

Non-practice owners mentioned that most practice owners were negative role models. Practice owners were reported to complain about tasks related to practice ownership and to have a poor work–life balance. These observations influenced the perceptions of non-practice owners, who mentioned that positive role models at a similar stage of life contributed to a positive perception of practice ownership.

#### Practices

Early in their careers non-practice owners have limited experience working in different practices, making it a challenge to determine their requirements for an ideal practice. This hinders short-term aspirations for practice ownership. The available practices that are seeking successors often do not align with the preferences of non-practice owners, further hampering steps towards ownership. By contrast, practice owners did not always find their ideal practice, but instead, adapted practices to fit their preferences. Choice of practice was influenced by their timeline for becoming a practice owner, the availability of practices, and the outcome of negotiations about acquisition.

#### Legislation

Legislation that requires freelance GPs *not* to work in a single practice for too long (that is, in a minimum of three practices per year) was mentioned by non-practice owners as a consideration for becoming a practice owner.

### Theme 4: Process

#### Prepared for practice ownership

All participants wanted to be better prepared for practice ownership, citing a lack of knowledge about the specific competences and skills required. They reported a need for more insights into the various aspects of practice ownership, such as practice organisation, tasks and roles, leadership, network organisations, finances, legislation, and practice acquisition, both during training and when working. However, some GP trainees questioned the feasibility of this additional training given the intensity of their current programmes. Freelance and salaried GPs considered practice management courses to be valuable, but they were concerned about prioritising them over existing commitments.

#### Process and approach

The decision to seek practice ownership requires that the various considerations informing this decision coincide favourably, which often takes time. Participants mentioned various issues in the process to becoming a practice owner. Unsuccessful takeovers and a lack of transparency about available practices hindered progress. Many non-practice owners preferred to wait for suitable opportunities or for a practice owner’s invitation, which prolonged the process. Additionally, participants suggested that having a coach could help them explore career options and the process of practice acquisition.

### Synthesis of themes and positive and negative considerations

Analyses of participants’ considerations about career decisions indicated that each individual has a complicated set of themes that can create positive or negative thoughts, and that these can change over time. Supplementary Table S2 integrates the themes and the positive and negative considerations. Overall, three sub-themes were predominantly positive and six were predominantly negative when considering practice ownership.

## Discussion

### Summary

This qualitative study explored the factors influencing Dutch GP decisions about practice ownership. Decisions reflect a complex interplay of professional, personal, external, and process-related factors. COC, autonomy, and personal development had predominantly positive influences on the decision to pursue practice ownership. By contrast, several factors had mainly negative influences, namely concerns about work–life balance, assuming ultimate responsibility, negative role models and a culture of complaint, practices not aligning with preferences, feeling unprepared for practice ownership, and dealing with issues in the process of becoming a practice owner. For GPs to choose practice ownership, numerous factors must coincide, and typically over a longer period. It was notable that non-practice owners felt that practice ownership could not be discussed and that practice owners did not recognise the social responsibility of non-owners.

### Strengths and limitations

A key strength of this research is the diversity of the sample, which included GP trainees, freelance and salaried GPs, practice owners, and ex-practice owners, providing insights into the considerations about practice ownership at different phases of a GP’s career. Data analysis and peer debriefing were improved by including a range of experts with different professional backgrounds.^34^ However, participant recruitment resulted in a sample with more women than in the national average for GPs aged 35–50 years (76%; national average, 68%).^35^ An overrepresentation of participants interested in career choices may also have occurred, which may mean that other considerations around practice ownership were missed. Finally, we included only a small number of ex-practice owners, and although no new topics emerged, we were unable to check this in additional interviews.

### Comparison with existing literature

Existing literature on considerations about practice ownership included enablers, namely autonomy, flexibility, and financial rewards. Autonomy was also recognised as an enabler in our study,^25^ whereas flexibility in work–life balance decisions was not for non-practice owners.^25^ Despite this, practice owners in our study did report more flexibility than they expected. This difference might be attributable to the size of Australian practices, with a larger number of GPs and staff members, which can offer greater flexibility than the typically smaller Dutch practices.^13,25^ Another factor could be the flexibility already experienced by freelance GPs in The Netherlands, which could minimise the difference between non-practice owners and practice owners. Although research has identified financial rewards as an enabler^23^ and financial concerns as a barrier,^23–25,27^ our participants did not consider these important, possibly owing to differing financial policies and income disparities between the countries.^28,36,37^

Additionally, our study identified COC as an enabler of practice ownership, aligning with a prior Dutch report,^14^ but not mentioned in previous literature. Personal development emerged as an enabler, especially for experienced GPs. This has not been described in the literature to date.

Previous studies^23–27^ identified five barriers to practice ownership, namely insufficient knowledge and skills, concerns about workload and work–life balance, financial concerns, increased responsibility, and bureaucracy. Three of these were also found in this study. First, insufficient knowledge and skills,^23–26^ labelled (un)preparedness in the present study, was a common issue in both countries. Second, the perceived workload and desire for a good work–life balance was mentioned as a barrier in previous research,^23–25,27,38^ with factors related to working as a GP and practice ownership mentioned as stressors that contribute to burnout.^38^ Third, increased responsibility,^25,27^ which we labelled ultimate responsibility, served as a barrier through linkage to pressures about work–life balance and fears that they may never be able to ’*switch off*’. The other two barriers, namely finances^23–25,27^ and bureaucracy^23^ were not mentioned in our study.

The following three additional barriers to practice ownership were identified: lack of positive role models; lack of suitable practices; and the process and approach. These additions may reflect differences between the healthcare systems and practices,^7,9,13,28–30^ which may lead to variations, such as how role models discuss practice ownership, the availability of practices, and the process of seeking a practice or successor.

Finally, we identified considerations that have not been mentioned previously and did not fit neatly into the enabler and barrier categories. These were social responsibility, task-related self-efficacy, settling in a region, and legislation.

**Figure 2.**
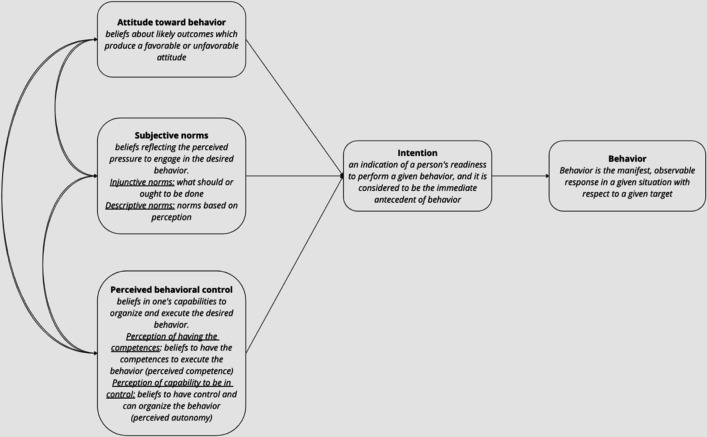
Model of theory of planned behaviour^41^

The theory of planned behaviour (TPB)^39–41^ can be used to explain how engagement in a particular behaviour, such as becoming a practice owner, is associated with attitudes towards that behaviour, subjective norms, and perceived behavioural control (Figure 2). For example, beliefs that practice owners have more COC, autonomy, and personal development contributed to a positive attitude, whereas belief that they had a disturbed work–life balance had a negative attitude, amplified by negative role models and apprehensions about having ultimate responsibility.

**Figure 3.**
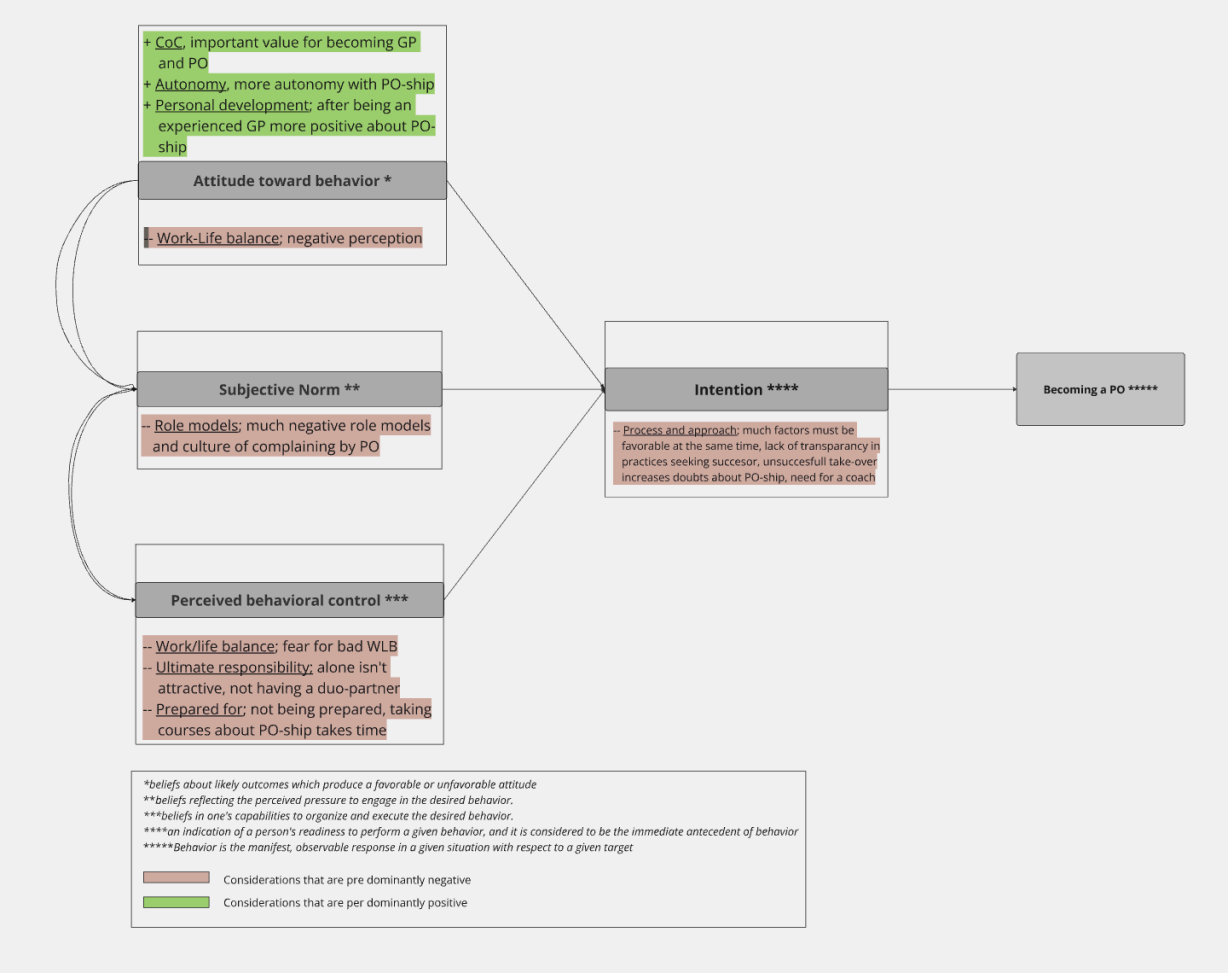
Model of theory of planned behaviour with predominantly positive and negative considerations for becoming a practice owner^41^

Our study showed that normative beliefs about social responsibility were positively linked to practice ownership, while descriptive norms in which the norm is exemplified by role models were associated negatively. Research shows that descriptive norms indirectly affect perceived behavioural control.^39^ Negative role models who complained about a work–life balance influenced the attitudes and perceived behavioural control of non-practice owners. Perceived control is crucial when forming intentions to engage in specific behaviours, even with positive attitudes and social pressure.^39^ Fear of loss of control as a practice owner, especially regarding their work–life balance, was a significant concern for non-practice owners. The prospect of taking on responsibility reinforces this fear and is unattractive (for example, particularly solo practices). It is unsurprising, therefore, that Dutch GPs felt unprepared for practice ownership.^41^ Despite non-practice owners recognising several positives of practice ownership, the combination of negative subjective norms and the lack of perceived behavioural control will hinder progress towards practice ownership. Practice owners could encourage prospective ownership through positive reinforcement, while lack of transparency in practices seeking a successor and unsuccessful takeovers will have negative impacts (Figure 3).

### Implications for research and practice

It is possible to enhance the sense of behaviour control with respect to the ability to succeed as a practice owner. This requires appropriate preparation for practice ownership, including understanding the required tasks and roles, practice organisation, developing entrepreneurial skills, and finding suitable partners. Allocating time to learn about practice ownership poses a challenge given competing demands from GP education, work commitments, and personal life.^7,26,27,38^ The key is to know when and under what conditions this preparation is most suitable. A coherent plan is needed of which a fellowship programme — that provides an opportunity for GPs to receive coaching on career choices and learn about practice ownership when it is being actively considered — can be a part.^26^ This approach should aim to improve perceptions about practice ownership and streamline the path to becoming a practice owner through appropriate preparation at a time when the GP is ready, thereby reducing the chance of dropout.

All GPs who are not practice owners need guidance regarding opportunities a practice owner has in managing their work–life balance, ideally from positive role models at the same life stage. It was disappointing to find that non-practice owners often perceive practice owners as negative role models who discouraged them from pursuing practice ownership.^42^ This issue could be addressed by complementing in-practice training that focuses on positive values^42–44^ with learning at a GP training centre to expose prospective owners to a wider variety of role models and a more comprehensive understanding of practice ownership.^23,24,27,39,43,44^ Efforts to raise awareness and shift culture among practice owners,^27^ so they offer a more balanced portrayal of practice ownership, will be vital to influencing subjective norms and perceived behavioural control toward practice ownership.

Becoming a practice owner is a complex process that raises questions about a wide range of alternatives to practice ownership, which fit the career aspirations and considerations of young GPs. Non-practice owners and practice owners share a sense of responsibility for COC. Unfortunately, non-practice owners also feel that discussions about alternatives to practice ownership are not possible and that they cannot freely discuss their negative considerations and hesitations with owners. It is essential to maintain open dialogue with inclusive participation among both groups, ensuring that COC is central to any discussion. The term ’patient ownership’, emphasising knowledge of and interaction with a patient group,^45^ may facilitate this discussion. Patient ownership should be explored further, incorporating the positive aspects of practice ownership such as autonomy and personal development. Including business expertise in the discussion may uncover possible approaches beyond the traditional structure of practice ownership. In addition, national policies should support developments to facilitate COC.

This study explored the factors that GPs consider when deciding whether to become a practice owner. Data from this study in the north of The Netherlands and from earlier studies can serve as a basis for future quantitative research, with the TPB providing a useful questionnaire for further measurement.^39,46^ It would be interesting to study the considerations for career choices among young GPs in other countries to determine universal and country-specific themes. A comparison between health systems in other countries, where GPs often work in a salaried setting, will provide insights into how the balance between practice ownership, GP employment, and freelance work influences COC. Finally, initiatives to tackle the current shortage of practice owners should be evaluated based on the results from this study and the TPB.

In conclusion, making career decisions about practice ownership is a complex process that crosses professional, personal, external, and process-related themes, with almost all (sub)themes having positive and negative considerations. Deciding in favour of practice ownership requires that multiple themes coalesce favourably at the same time. Integrating the TPB provided added insight into the complexity of the problem; the implications for future practice and research. To establish practice ownership, perceived behavioural control and subjective norms must change. Practice owners and non-practice owners must engage in open discussions that evaluate practice ownership and consider other models.

## Data

The dataset relied on in this article is available from the corresponding author on reasonable request.
